# Clinical outcomes of laser-assisted non-surgical periodontal therapy for chronic periodontitis treatment

**DOI:** 10.6026/973206300212789

**Published:** 2025-08-31

**Authors:** Santosh Kumar Verma, Sharmila Kumari, Neha Kumari, Mayank Priyadarshi, Ankita Verma, Harsh Priyank, Vivek Gupta

**Affiliations:** 1 Department of Periodontology and Oral Implantology, Dental Institute, Rajendra Institute of Medical Sciences, Ranchi, India; 2 Department of Pedodontics and Preventive Dentistry, Hazaribagh College of Dental Sciences and Hospital, Hazaribagh, India; 3 Department of Conservative Endodontics and Aesthetic Dentistry, Dental Institute, Rajendra Institute of Medical Sciences, Ranchi, India

**Keywords:** Chronic periodontitis, diode laser, non-surgical periodontal therapy, clinical outcomes, periodontal pocket

## Abstract

Chronic periodontitis remains a prevalent oral disease that leads to progressive tissue destruction, and conventional scaling and root planing alone often provide limited healing. Therefore, it is of interest to assess the efficacy of the diode laser as an adjunct to scaling and root planing in the treatment of chronic periodontitis. 50 patients were treated on one side with scaling and root planing combined with the application of diode laser therapy (Group A), while the contralateral side received scaling and root planing alone (Group B). Clinical parameters included clinical attachment level (CAL), plaque index (PI), bleeding on probing (BOP), probing depth (PD) and gingival index (GI), which were recorded at baseline, 1 and 3 months post-treatment, respectively. The combined therapy group (Group A) showed notable improvements in CAL, GI, BOP and PD and confirmed that diode laser therapy enhances clinical outcomes and promotes faster healing in the management of chronic periodontitis.

## Background:

Periodontitis is known as one of the most common oral diseases worldwide; chronic periodontitis (CP) is characterized by inflammation caused by bacterial infection, which leads to the creation of periodontal pockets, gradual loss of periodontal support and the risk of tooth loss in susceptible individuals [1]. The close association between bacterial plaque and chronic periodontitis makes the removal of bacterial deposits a primary objective of the periodontal therapy [2]. Periodontal therapy primarily aims to halt the inflammation by reducing the number of pathogenic microorganisms in the periodontal tissues. Successfully eliminating supragingival and subgingival biofilms, the smear layer (containing bacteria, endotoxins and contaminated cementum), is crucial to this treatment success [3, 4]. Eliminating these harmful substances is essential for achieving biological compatibility between the affected periodontal root surface and the new connective tissue attachment. Conventional non-surgical periodontal treatment, the primary recommended method, involves SRP for patients with untreated periodontitis. This approach aims to clean the contaminated root surfaces and eliminate the etiological factors from both the supra- and sub-gingival areas of the tooth, along with the surrounding inflamed tissues using hand instruments and ultrasonic scalers [5, 6]. Many systematic reviews indicate similar improvements in clinical parameters with hand and ultrasonic instrumentation, yet some researchers favor ultrasonic root debridement for its efficiency [7]. Additionally, ultrasonic instrumentation requires less time and physical effort than manual methods [8]. However, completely remove bacterial biofilm and endotoxins from deeper pockets and furcation sites remains challenging with both techniques [9]. Incomplete removal of subgingival calculus and bacterial deposits from the root surface can compromise treatment effectiveness, leading to unsatisfactory results. To address these limitations, various adjunctive techniques have been proposed, including laser radiation [10]. Previous randomized clinical trials have demonstrated the potential benefits of laser-assisted non-surgical periodontal therapy in improving clinical parameters and reducing inflammation [11]. These supplementary methods aim to enhance the removal of residual pathogens and improve overall treatment outcomes in difficult-to-reach areas.

Laser therapy has emerged as a viable alternative or supplementary treatment to traditional periodontal therapy. Comprehensive reviews summarize that laser-assisted non-surgical treatments can enhance clinical outcomes in periodontitis by targeting bacterial biofilms and promoting tissue healing, though clinical efficacy varies by laser type and protocol [12]. The bactericidal and detoxifying effects of the diode laser in non-surgical periodontal treatment have been extensively documented in numerous studies. In patients with chronic periodontitis, the combination of diode laser therapy with SRP yields superior outcomes compared to either SRP or laser treatment alone, especially regarding microbial reduction and clinical parameters [13]. Moritz *et al.* (1998) observed a notable decrease in bacterial load and inflammation when diode laser therapy was combined with SRP [14]. Notwithstanding encouraging outcomes, there remains a persistent debate regarding the effectiveness of laser therapy in periodontal treatment. Certain studies suggest that the combination of laser therapy with SRP yields no substantial advantages in microbiological outcomes or inflammation reduction. A systematic review by Karlsson *et al.* emphasized the insufficiency of studies regarding the clinical effects of laser therapy in conjunction with SRP [15, 16]. Therefore, it is of interest to assess the effectiveness of laser therapy as a supplementary treatment to nonsurgical periodontal therapy in patients with chronic periodontitis and to guide treatment strategies for individuals in the maintenance phase of periodontal care.

## Materials and Methods:

A randomized controlled split-mouth technique was conducted on 50 systemically healthy subjects aged between 25-60 years in the Dental Institute, Rajendra Institute of Medical Sciences (RIMS), Ranchi. The study was conducted over a period of 6 months. Fully co-operative, willing subjects with no periodontal treatment from the last six months, with a relative attachment level of 3mm and a pocket depth of 4mm or more at one or more sites were selected for the study. Pregnant and/or lactating females, smokers and subjects using antimicrobials and analgesics of any form were not included in the study. Subjects with less than 16 teeth, teeth with grade III mobility or pockets with greater than 10mm and subjects with partial or fixed prosthesis were also excluded from the study. Patients were free to leave the study at any point during the treatment with no major complications or discussions. Before the initiation of the study, institutional ethical clearance was obtained. A brief discussion with each subject about the study's purpose, time commitment and benefits of the treatment was conducted. After the complete agreement of the subjects, an informed consent form available in both Hindi and English was signed by the patients. To prevent bias, study details were kept blinded from the operator. Demographic information of patients, encompassing age, gender and socio-economic status, was gathered. The patients were seated comfortably in a well-lit dental chair and a comprehensive medical, dental and antimicrobial history was obtained. Every patient received a comprehensive dental assessment, encompassing any prior periodontal interventions. Clinical parameters were evaluated utilizing a Michigan 0 probe with William markings. The study was completed in three months and involved evaluations at baseline, one month and three months post-initiation. Subjects selected for the participation of the study were first assessed for the clinical parameters by the second single operator. Clinical parameters, including CAL, BOP, PI, PD and GI, were measured and recorded for each patient in their patient data record sheet. The arches of 50 subjects were divided into two quadrants without any knowledge of the operator (third) and patients were coded with numbers and letters and randomization of quadrants was done. Subsequently, the two quadrants were subdivided into two: one moderate pocket with a pocket depth of 4-6mm and moderate attachment loss of 7-9mm and a second deep pocket with a pocket depth of 7mm and attachment loss of more than 10mm. Patients in Group A received diode laser treatment alongside SRP, while Group B underwent SRP with piezo scalers only. The groups were designed to prevent bias when recording clinical parameters at one and three months. All patients were given oral hygiene instructions and received reinforcement during follow-up visits. A comprehensive subgingival SRP was conducted in a single session under local anesthesia (if necessary) for each patient in both groups, utilizing piezoelectric scalers by uninformed clinicians. In Group A, adjunct laser therapy was administered twice, on the first and seventh days post-scaling, by a different clinician. The laser therapy employed a Gallium-aluminum arsenide diode laser with a wavelength of 940 nm, a power output of 0.66W and an energy density of 15 j/cm^2^, utilizing a fiber optic delivery system in pockets with sweeping motions for 30 seconds per tooth. Pocket irrigation was conducted consistently following each treatment session.

## Statistical analysis:

The measurements recorded were then grouped according to the study and transferred to Microsoft Excel for proper data analysis. The grouped data were then analyzed using SPSS software, version 21.0, to obtain results. The differences between the values of clinical parameters, including PI, GI, BOP, PD and CAL, were evaluated using the mean. Changes in pocket depth and clinical attachment level were evaluated using the initial and the final values. Mann-Whitney U and Wilcoxon test and chi-square test were used for the analysis and the data were represented graphically using bar diagrams. Bonferroni corrections were made, as the analysis of the two groups was done at three different time intervals. Pocket depth and Relative attachment level analysis were done by using the chi-square test and a p-value < 0.05 was considered significant.

## Results:

Out of the total 50 patients, 68% were males and only 32% were females, with a mean age of 49.020±10.289 years, diagnosed with chronic periodontitis. The maximum population of the study belonged to the upper middle class (34%) of the urban area (1.600±0.070). 30% of the population belonged to the upper lower, 20% to the lower middle, 10% to the upper class and only 3% belonged to the lower socio-economic class (Table 1 - see PDF, Figure 1 - see PDF). For Plaque Index, in Group A (SRP + diode laser application), the mean ± Standard deviation at baseline was 1.843 ±0.149, after 1 month, it was found to be 0.733±0.301 and after 3 months, it was found to be 0.540±0.230. In Group B (SRP) at baseline, a mean and standard deviation of 1.570 ±0.404, after 1 month 0.526±0.298 and after 6 months, 0.530 ± 0.530±0.290 were found among the study patients. The mean difference in Group A from baseline to 1 month was -1.110 with a p-value of <0.0001, from 1 month to 3 months was -0.193 with a p-value of 0.0005 and from baseline to 3 months was -1.303 with a p-value of <0.0001. In Group B (SRP), the mean difference from baseline to 1 month was -0.720 with a p-value of <0.0001, from 1 month to 3 months was -0.270 with a p-value of <0.0001 and from baseline to 3 months was -0.990 with a p-value of <0.0001 (Table 2 - see PDF, Figure 2 - see PDF). For Gingival Index, In Group A (SRP + diode laser application), the mean ± Standard deviation at baseline was 1.869 ±0.302, after 1 month, it was found to be 0.740±0.278 and after 3 months, it was found to be 0.350±0.230. In Group B (SRP) at baseline, a mean and standard deviation of 1.530 ±0.541, after 1 month 0.733±0.301 and after 3 months 0.310±0.180 was found among the study patients. The mean difference in Group A from baseline to 1 month was -1.129 with a p-value of <0.0001, from 1 month to 3 months was -0.390 with a p-value of <0.00001 and from baseline to 3 months was -1.519 with a p-value of <0.0001. In Group B (SRP), the mean difference from baseline to 1 month was -0.797 with a p-value of <0.0001, from 1 month to 3 months was -0.423 with a p-value of <0.0001 and from baseline to 3 months was -1.220 with a p-value of <0.0001 (Table 2 - see PDF, Figure 2 - see PDF). In group A (SRP + diode laser application), the reduction in the percentage of bleeding sites from baseline to 1 month was 53.3%, from baseline to 3 months was 60% and from 1 month to 6 months was -6.7%. In group B (SRP), the reduction in the percentage of bleeding sites from baseline to 1 month was 66.7%, from baseline to 3 months was 68.7% and from 1 month to 6 months was -2% (Table 3 - see PDF, Figure 3 - see PDF). In Tables 4 (see PDF) and 5 (see PDF), non-significant results can be seen when the mean values were compared from 1 month to 3 months, respectively. In case of moderate attachment loss, in Group A (SRP + diode laser application), a mean difference of -0.530 (p=0.9500) was obtained while in Group B (alone SRP), a mean difference of -1.430 (p=0.2185) was obtained. On evaluating the results of patients with severe attachment loss, in Group A, a mean difference of -0.880 (p=0.0318) was obtained when measured 1 month to 3 months, while in Group B, a value of -0.990 (p=0.085) was obtained. In Group A (SRP + diode laser application)with moderate pocket depth of 4mm to 6mm, a mean difference of -0.380 (p=0.223) was obtained after 1 month and 3-month mean value evaluation, while in Group B (alone SRP), a mean difference of -0.580 (p=0.5001) was obtained. In Group A (SRP + diode laser application), with a pocket depth of more than 7mm, a mean difference of -0.010 (p=0.9694) was obtained, while in Group B, a mean difference of -0.168 (p=0.6612) was obtained (Tables 4 and 5 - see PDF).

## Discussion:

Non-surgical laser therapy has been studied as an adjunct to conventional periodontal treatments, with promising results in some areas, but with limited clinical evidence confirming its overall benefit [17]. Lasers, particularly diode lasers at wavelengths of 805 nm or 940 nm, may enhance SRP by improving subgingival debridement and reducing harmful microorganisms, potentially facilitating connective tissue attachment and promoting periodontal healing. Studies have shown that the 805-nm diode laser may enhance subgingival debridement and reduce bacteria in periodontal pockets over 4 mm, while the 940-nm laser may influence growth factor expression in gingival fibroblasts [18]. However, clinical evidence is insufficient to substantiate significant advantages of laser therapy over conventional periodontal treatments [19]. The findings of our study indicate that non-surgical periodontal treatment, utilizing piezo instruments either independently or alongside a diode laser, led to substantial enhancements in clinical metrics, including BOP, PD and CAL for both moderate and deep pockets at one and three months following treatment. Prior studies have demonstrated that traditional SRP alongside oral hygiene guidance is effective in managing and controlling CP. The present study utilized a 980-nm diode laser with a power output of 0.66 Was an adjunct to SRP to significantly diminish gingival inflammation throughout the observation period. Nevertheless, the results did not indicate that the laser provided superior outcomes compared to SRP alone. This finding aligns with the research conducted by Assaf *et al.* which revealed no additional therapeutic advantages of the diode laser on gingival healing [15]. Our study revealed that both Group A and Group B exhibited favorable post-treatment results; however, the laser group demonstrated more significant enhancements in probing depth reduction and clinical attachment level gain, especially for moderate pockets, from baseline to 3 months. Nonetheless, there were no substantial differences in outcomes between 1 month and 3 months, suggesting that although the diode laser provided certain benefits, its superiority over SRP alone for gingival healing and inflammation was not definitively established. Aykol *et al.* (2011) conducted a study revealing that the gallium-aluminum-arsenide diode laser, employing a non-contact biostimulation technique, resulted in a more significant reduction in probing depth in moderate pockets at 1, 3 and 6 months post-periodontal therapy [20]. This study yielded comparable results, indicating that diode laser therapy is more efficacious in moderate pockets. De Micheli *et al.* (2011) found no significant enhancements in clinical or microbiological parameters six weeks post high-power diode laser therapy combined with SRP. This discrepancy may be attributed to the frequency of laser application (twice in De Micheli's study compared to three times in the present study) [21]. Lai *et al.* (2009) observed no significant differences in clinical or radiographic outcomes between laser-treated and control sites after several months of low-power helium-neon laser application, corroborating the current study's findings that the diode laser did not yield substantial clinical enhancements [22]. Kamma *et al.* (2013) also noted that combining diode laser therapy (980 nm) with SRP showed greater benefits in both clinical and microbiological measures, particularly in patients with aggressive periodontitis, compared to SRP or laser therapy alone [23]. Recent studies demonstrate that laser treatments, when combined with traditional non-surgical periodontal therapy, produce only modest clinical enhancements, with discrepancies observed among the research findings. A possible explanation for the reduced efficacy of laser therapy in infected periodontal pockets is the substantial reduction in laser power output at the optical fiber tip, which can be alleviated by routinely cleaving the tips [24]. To ensure the proper energy delivery by the laser, De Micheli *et al.* recommended using a power meter to measure the actual energy delivered by a laser, as the displayed energy on the device may not reflect the true energy at the fiber-optic tip due to transmission losses [21]. There is little consensus on clinical outcomes, even among studies using the same laser wavelength, due to small sample sizes and inconsistent descriptions of disease severity. This variability complicates meta-analyses and the comparison of results across different research studies [16]. A randomized controlled clinical trial conducted by Caruso *et al.* demonstrated modest enhancements in clinical parameters within the laser group when a diode laser was utilized as an adjunct to scaling and root planing (SRP). Nonetheless, the study's restricted sample size (19 teeth from 13 patients) complicates the ability to reach definitive conclusions [25]. Standardized criteria for periodontal laser therapy are needed to establish guidelines on energy, application time, irradiation modes, power settings and laser types. Establishing such standards would enhance consistency, comparability and reliability in future studies, facilitating cross-study comparisons.

## Conclusion:

The lasers have a limited impact as an addition to conventional periodontal therapies, with inconsistent results due to varying laser settings and methods. It emphasizes the need for more randomized controlled trials with larger sample sizes and standardized protocols to clarify the effectiveness of laser therapy in periodontal care.

## References

[1] Meyle J, Chapple I. (2015). Periodontol 2000..

[2] Koyanagi T (2013). J Clin Periodontol..

[3] Cugini MA (2000). J Clin Periodontol..

[4] Goldman HM (1948). J Periodontol (1930)..

[5] Mombelli A (2018). Periodontol 2000..

[6] Sherman PR (1990). J Periodontol..

[7] Oda S (2004). Periodontol 2000..

[8] Kocher T (2005). J Clin Periodontol..

[9] Copulos TA (1993). J Periodontol..

[10] Caffesse RG, Echeverría JJ. (2019). Periodontol 2000..

[11] Everett JD (2017). Open Dent J..

[12] El Mobadder M (2023). Encyclopedia..

[13] Miyazaki A (2003). J Periodontol..

[14] Moritz A (1997). J Clin Laser Med Surg..

[15] Assaf M (2007). Photomed Laser Surg..

[16] Karlsson MR (2008). J Periodontol..

[17] Cobb CM (2006). J Periodontol..

[18] Hakki SS, Bozkurt SB (2012). Lasers Med Sci..

[19] Slot DE (2009). J Periodontol..

[20] Aykol G (2011). J Periodontol..

[21] De Micheli G (2011). Lasers Med Sci..

[22] Lai SM (2009). Photomed Laser Surg..

[23] Kamma JJ (2009). Photomed Laser Surg..

[24] Dukic W (2013). J Periodontol..

[25] Caruso U (2008). New Microbiol..

